# Dose evaluations of organs at risk and predictions of gastrointestinal toxicity after re-irradiation with stereotactic body radiation therapy for pancreatic cancer by deformable image registration

**DOI:** 10.3389/fonc.2022.1021058

**Published:** 2023-01-30

**Authors:** Yangsen Cao, Xiaofei Zhu, Chunshan Yu, Lingong Jiang, Yongjian Sun, Xueling Guo, Huojun Zhang

**Affiliations:** Department of Radiation Oncology, Changhai Hospital Affiliated to Naval Medical University, Shanghai, China

**Keywords:** pancreatic cancer, stereotactic body radiation therapy, dose distributions, re-irradiation, organs at risk (OARs)

## Abstract

**Purpose:**

Re-irradiation of locally recurrent pancreatic cancer may be an optimal choice as a local ablative therapy. However, dose constraints of organs at risk (OARs) predictive of severe toxicity remain unknown. Therefore, we aim to calculate and identify accumulated dose distributions of OARs correlating with severe adverse effects and determine possible dose constraints regarding re-irradiation.

**Methods:**

Patients receiving two courses of stereotactic body radiation therapy (SBRT) for the same irradiated regions (the primary tumors) due to local recurrence were included. All doses of the first and second plans were recalculated to an equivalent dose of 2 Gy per fraction (EQD_2_). Deformable image registration with the workflow “Dose Accumulation-Deformable” of the MIM^®^ System (version: 6.6.8) was performed for dose summations. Dose–volume parameters predictive of grade 2 or more toxicities were identified, and the receiver operating characteristic (ROC) curve was used to determine optimal thresholds of dose constraints.

**Results:**

Forty patients were included in the analysis. Only the *V*_10_ of the stomach [hazard ratio (HR): 1.02 (95% CI:1.00–1.04), P = 0.035] and *D*_mean_ of the intestine [HR: 1.78 (95% CI: 1.00–3.18), P = 0.049] correlated with grade 2 or more gastrointestinal toxicity. Hence, the equation of probability of such toxicity was $\text{P}=\frac{1}{1+e^{-\left(-4.155+0.579D\text{mean of the intestine}+0.021\text{V}10\text{ of the stomach}\right)}}$ Additionally, the area under the ROC curve and threshold of dose constraints of *V*_10_ of the stomach and *D*_mean_ of the intestine were 0.779 and 77.575 cc, 0.769 and 4.22 Gy_3_ (α/β = 3), respectively. The area under the ROC curve of the equation was 0.821.

**Conclusion:**

The *V*_10_ of the stomach and *D*_mean_ of the intestine may be vital parameters to predict grade 2 or more gastrointestinal toxicity, of which the threshold of dose constraints may be beneficial for the practice of re-irradiation of locally relapsed pancreatic cancer.

## Introduction

Despite the advances of modality and treatment regimens, pancreatic cancer still remains a lethal disease with a low survival rate and increasing mortality (1). Similar findings were also identified in China (2). Although surgical resection is considered as a curative option, only less than 20% of patients were candidates for up-front surgery at the initial diagnosis. Hence, chemoradiotherapy may be an alternative for most patients with advanced pancreatic cancer. However, a significant number of patients would still develop local recurrences within the primary regions after aggressive treatment. Those patients may not be amenable to surgery or second-line chemotherapy due to the high incidences of perioperative complications or chemotherapy-induced toxicities (3–5). A second radiotherapy may be employed with caution at the physician’s discretion. In the case of radiotherapy technique, stereotactic body radiation therapy (SBRT) has commonly been used in locally advanced pancreatic cancer. Additionally, previous studies have clarified the feasibility of delivery of re-irradiation with SBRT for pancreatic cancer (6–10).

Regarding retreatment, it is a challenge to achieve good local control with proper radiation doses without compromise of protection of organs at risk (OARs), namely, keeping the doses under desired limits. Moreover, no standards about the dose constraints in the second radiotherapy have been proposed. Hence, in clinical practice, dose evaluations of the normal tissues might depend on dose distributions in the first treatment projecting to those in the second radiotherapy *via* image registration, which resulted in direct dosimetric comparisons of the plans other than assessment based on biological quantities. Only maximum doses to OARs may be converted into equivalent dose in 2 Gy/f and summed by the linear quadratic model (11).

Additionally, the fusion of images from the first and second radiotherapy for dose summations and evaluations was an obstacle of precise delivery, especially for SBRT. Typically, the rigid image registration (RIR) was employed for registrations of the first images with the second one, where the processed translation and rotation of the first images were compromised to be aligned with the second images. Most SBRT systems are equipped with the RIR, although the deformable image registration (DIR) has been developed. However, in the case of re-irradiation, due to gastrointestinal motility, tumor growth, or changes in the patient’s weight, discrepancies between the alignment of the first and second images may contribute to the inaccurate evaluations of summed doses albeit calculated with the RIR.

In this scenario, the DIR provides both geometric and dosimetric accuracy compared to the RIR, which is pivotal to map, overlap, and integrate information from different images. As a result, quantifications of summed doses to OARs over the courses of treatment could be achieved by doses mapped back to a common reference anatomy with the DIR (12–15). Dose accumulations are calculated by warping dose grids to the reference anatomy based on the obtained deformation vector field (12–14).

Limited studies have investigated re-irradiation with SBRT for pancreatic cancer, which demonstrated high local control with a 1-year rate of 62%–81% and acceptable toxicities (6–8, 16, 17). Nonetheless, no further studies have evaluated accumulated doses to OARs and the correlation between doses and toxicities so far. Additionally, previous studies about re-irradiation all adopted conventional radiotherapy as the first treatment. Therefore, the aim of the study was to calculate accumulated dose distributions of OARs from two courses of SBRT and identify the correlations between radiation-induced toxicities with the doses to OARs, which might provide evidence for the determination of potential acceptable dose constraints for re-irradiation with SBRT.

## Methods

### Eligibility

From 2012 to 2017, patients with biopsy- and radiographically proven pancreatic cancer who received two courses of SBRT were screened for eligibility. Patients undergoing the second SBRT for other targets other than the primary lesions were excluded from the study. A total of 40 patients received two courses of SBRT for the same irradiated regions (the primary tumors) due to local recurrence.

### Dose constraints

The baseline dose constraints referred to TG-101 (18). The maximum dose of the OAR was calculated as 50% more than the normal constraint in the case of re-irradiation. Due to different doses to target regions and OARs and fractionation schemes, all treatment schedules were recalculated to an equivalent dose of 2 Gy per fraction (EQD_2_) based on the following formula: $EQD_{2}=d*n*\frac{d+\frac{\alpha }{\beta }}{2+\frac{\alpha }{\beta }}$ An α/β value of 10 Gy (Gy_10_) was employed for the tumor dose and acute effects, and the value determined as 3 Gy (Gy_3_) concerns late effects. Secondly, the correlation between dose attenuation and time interval between two courses of radiotherapy was based on a previous study (120). Therefore, we allowed a dose reduction of 50% of the first radiation dose to OARs as the baseline for a re-irradiation 12 months after the last radiation. A dose reduction of 25% of the first radiation dose to OARs as the baseline was allowed for re-irradiation after 6–12 months. No dose reduction was used when re-irradiation was done within 6 months (19).

### Treatment planning

The protocol of SBRT was similar to our previous studies (20–22). SBRT was delivered *via* CyberKnife^®^ (Accuray Incorporated, Sunnyvale, CA, USA). Three to five gold fiducials within or adjacent to the pancreatic tumor were preferable. A radiographically evident gross disease was regarded as the gross tumor volume (GTV). The clinical target volume (CTV) was defined as areas of the potential subclinical disease spread. In most cases, the CTV was equal to the GTV. The planning target volume (PTV) included a 2–5-mm margin on the GTV. The dose was prescribed to the 70%–80% isodose line, covering at least 90% of the PTV. However, doses would be reduced at the physician’s discretion if the tumor was located one-third or more to the duodenum or stomach circumference, or if the tumor abutted the bowel in only one area, as determined by the relationship of the tumor to the duodenum in axial, coronal, and sagittal planes in CT scans, or if the distance between the tumor and the bowel wall less than 3 mm.

### Dose summation

**Figure 1:**
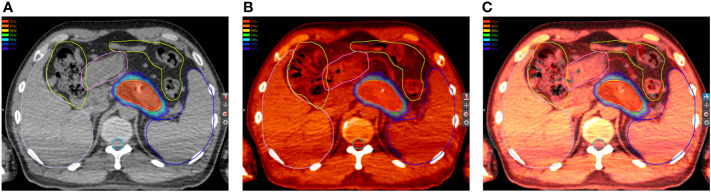
Organs at risk (OARs) in the **(A)** first and **(B)** second plan. **(C)** OARs in the first plan projected to the second plan.

**Figure 2:**
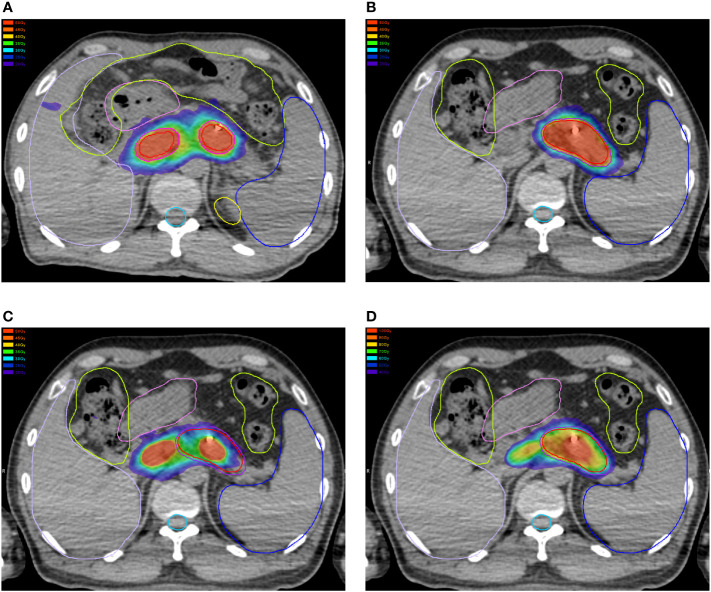
Dose distributions in the **(A)** first and **(B)** second plan. **(C)** Dose distributions in the first plan projected to the second CT scans. **(D)** Summations of dose distributions of the first and second plan by deformable image registration.

Delineations of OARs depended on the treatment schemes. The liver, stomach, duodenum, and kidneys were contoured completely. The esophagus and bowels were contoured based on the extent of radiation fields of the two treatment plans, and the volumes should coincide. The difference of volumes of the duodenum, stomach, and bowel between the two plans should be less than 15, 30, and 80 cm^3^, respectively. Hence, the OARs in the first plan were required to be the same as those in the second plan (**Figure 1**). Dose distributions, structures sets, and CT scans of the two treatment plans were extracted from the Multiplan^®^ System (version: 4.0.2) and sent to the MIM^®^ System (version: 6.6.8) for analysis. Firstly, two CT scans were aligned rigidly *via* automatic bone matches (translation and rotations). Therefore, for each plan before summation, each of the contoured OARs was registered rigidly. Subsequently, the DIR with the workflow “Dose Accumulation-Deformable” of MIM was performed for dose summations, which has been used in dose distributions of other cancers (23–25). After the DIR, the dose distributions of the first plan were projected to the second treatment with both doses converted to EQD_2_, which were summed up finally (**Figure 2**). The modifications of image fusions with the DIR were performed by Reg Refine and Reg Reveal, the quality control modes in MIM. Afterward, the dose–volume histograms (DVHs) were derived from the summed plans of patients with two courses of SBRT by summed dose distributions. The OARs with maximum doses exceeding the redefined dose constraints were selected, and the correlations between excessive doses and toxicities were reanalyzed. The overlap target volume was defined as the volume covered by 95% isodose line of the summed dose.

### Quality assurance of the deformable image registration (DIR)

Reg Reveal and Reg Refine are the primary tools used to evaluate and adjust a deformation in order to achieve accurate results. Reg Reveal was developed for this purpose and is currently the only tool available for the specific purpose of efficient quality assurance (QA) of the DIR. Reg Reveal allows the user to interrogate the registration in specific regions of interest and draw conclusions about its accuracy. Reg Refine allows the user to influence the registration algorithm to achieve a more accurate result.

Reg Refine is an input into multiple DIR algorithms. It allows the user to define local rigid alignments to provide additional information to help guide the deformation algorithm near these areas. It can be used iteratively to execute a DIR, evaluate the local DIR accuracy, and suggest local alignments to improve the DIR result until an optimal alignment is achieved. The DIR was first evaluated with Reg Reveal to determine areas of the registration that needed improvement. Rigid registration adjustment tools were then used in areas where the naive DIR was determined to be inaccurate to allow the observer to manually adjust the local registration or to execute an automatic rigid registration within a box of interest. The observer then recorded this preferable local alignment. When re-executing the DIR, these recorded local rigid alignments were used as inputs to influence the algorithm to achieve a local DIR closer to this observer-defined result.

### Toxicity and efficacy

The toxicity of treatment was evaluated in detail and scored for each patient. In addition, the efficacy of two courses of SBRT was assessed based on the tumor response, amelioration of pain, improvement of quality of life, and gain of weight during follow-up. Acute toxicities were determined using the “Acute radiation morbidity scoring criteria” from the Radiation Therapy Oncology Group, while late toxicities were evaluated using the “Late radiation morbidity scoring schema” from the Radiation Therapy Oncology Group/European Organization for Research on the Treatment of Cancer (26).

### Statistical analysis

The correlation between doses and toxicities was determined by logistic regression (backward conditional), where the potential dose–volume parameters predictive of toxicities were identified. The goodness of fit of the logistic model was analyzed using the Hosmer–Lemeshow test. The optimal risk threshold of each predictor was determined by the receiver operating characteristic (ROC) curve. Afterward, the probability of each patient developing gastrointestinal toxicity derived from predictors in the logistic regression analysis was also analyzed with the ROC curve to identify the optimal thresholds of probability. Two-sided P values<0.05 were considered statistically significant. Statistical analyses were performed using SPSS version 22.0 (IBM Corporation, Armonk, NY, USA).

## Results

### Dose distributions

Patients’ characteristics were demonstrated in **Table 1**. The median time interval of the two courses of SBRT was 11.4 months (range: 3.8–29.1 months). The median prescription dose of the initial and second courses of SBRT was 35.5 Gy/5–7f and 32 Gy/5–8f, respectively. The median EQD_2_ of PTV in the first and second SBRT was 49.58 Gy_10_ (range: 40 Gy_10_–71.25 Gy_10_) and 41.85 Gy_10_ (range: 31.25 Gy_10_–55.73 Gy_10_), respectively. Details were shown in **Table 2**. The accumulated doses of the OARs, including the stomach, duodenum, bowel, liver, spinal cord, and kidneys were demonstrated in **Table 3**.

**Table 1:** Patient characteristics.

Characteristic	(n=40)
Age (years)	60.7 ± 10.9
Sex	
Male	28
Female	12
T category	
T1	1
T2	7
T3	8
T4	24
Pancreas location	
Head and neck	25
Body and tail	15

**Table 2:** Prescription doses to the PTV.

	First SBRT	Second SBRT
Prescription doses	35.5 Gy (30-46.8 Gy)	32.25 Gy (25-38 Gy)
EQD_2_	49.58 Gy_10_ (40-71.25 Gy_10_)	41.85 Gy_10_ (31.25-55.73 Gy_10_)
PTV	37.75 cc (10.70-196.07 cc)	23.62 cc (8.81-278.42 cc)

EQD2, equivalent dose in 2Gy per fraction; PTV, planning target volume.

**Table 3:** Accumulated doses after the deformable image registration.

OARs	Dose	First radiation	Re-irradiation	Summed doses	Summed doses based on the correlation of dose attenuation and time
Stomach	*D*_max_(Gy_3_)	30.20 (3.18-55.27)	18.89 (3.01-44.25)	43.25 (7.56-90.47)	36.75 (5.79-76.74)
	*D*_1cc_(Gy_3_)	22.24 (2.28-34.78)	14.83 (2.57-35.18)	35.08 (4.88-76.88)	27.86 (4.30-65.69)
	*D*_10cc_(Gy_3_)	15.27 (2.10-25.11)	9.87 (1.79-1.05)	24.59 (3.49-60.72)	19.78 (2.97-50.80)
	*D*_mean_(Gy_3_)	5.02 (0.65-9.96)	3.07 (0.67-6.83)	7.97 (1.55-13.38)	6.42 (1.33-10.98)
	*V*_10_(cm^3^)	36.57 (0-154.05)	9.37 (0-71.38)	99.69 (0-337.81)	66.41 (0-202.16)
	*V*_20_(cm^3^)	2.66 (0-32.01)	0.01 (0-12.04)	22.73 (0-121.58)	7.69 (0-111.30)
	*V*_30_(cm^3^)	0.04 (0-3.39)	0 (0-2.02)	3.59 (0-43.95)	0.40 (0-46.50)
Duodenum	*D*_max_(Gy_3_)	24.43 (1.67-51.53)	15.45 (1.55-35.11)	35.61 (3.12-73.62)	30.36 (2.74-58.23)
	*D*_1cc_(Gy_3_)	18.18 (1.29-28.45)	11.00 (0.81-20.78)	26.82 (2.58-63.70)	22.13 (2.27-39.85)
	*D*_5cc_(Gy_3_)	12.44 (1.06-21.31)	7.04 (0.70-17.98)	20.77 (2.30-60.31)	15.99 (2.03-37.96)
	*D*_10cc_(Gy_3_)	9.58 (1.03-18.95)	5.34 (0.69-16.40)	16.26 (1.99-57.25)	12.82 (1.73-35.69)
	*D*_mean_(Gy_3_)	5.10 (1.02-12.58)	2.81 (0.68-9.25)	7.75 (1.93-23.75)	6.18 (1.63-19.59)
	*V*_10_(cm^3^)	9.05 (0-48.85)	1.47 (0-71.86)	23.26 (0-176.92)	18.66 (0-151.23)
	*V*_20_(cm^3^)	0.50 (0-7.52)	0 (0-1.71)	5.37 (0-112.89)	1.72 (0-56.03)
	*V*_30_(cm^3^)	0 (0-0.72)	0 (0-0.13)	0.56 (0-48.44)	0.05 (0-4.20)
Intestine	*D*_max_(Gy_3_)	30.77(17.51-43.46)	20.64(10.89-37.48)	44.07(29.70-92.47)	35.76(22.04-63.17)
	*D*_1cc_(Gy_3_)	23.95(13.50-33.21)	16.20 (8.51-29.63)	35.25(20.99-74.75)	28.06(17.28-46.80)
	*D*_5cc_(Gy_3_)	19.94(11.11-28.38)	13.41 (6.64-25.71)	28.86(16.89-43.45)	22.54(13.05-39.69)
	*D*_mean_(Gy_3_)	2.92 (0.90-8.28)	2.10 (0.97-5.63)	5.23 (2.04-14.04)	4.03 (2.15-11.64)
	*V*_20_(cm^3^)	4.93 (0-88.89)	0.07 (0-25.98)	31.91(1.42-309.13)	8.56 (0.01-109.21)
	*V*_30_(cm^3^)	0.07 (0-3.14)	0 (0-0.86)	5.02 (0-67.70)	0.30 (0-25.42)
Spinal Cord	*D*_max_(Gy_3_)	5.62 (1.43-14.54)	3.41 (1.04-16.92)	8.51 (3.61-18.88)	6.43 (3.07-17.36)
	*D*_0.35cc_(Gy_3_)	5.07 (1.38-13.34)	3.03 (0.93-13.64)	7.83 (3.47-16.25)	5.83 (2.69-14.17)
Left Kidney	*D*_mean_(Gy_3_)	3.18 (0.53-11.62)	2.16 (0.72-8.78)	5.47 (1.21-17.56)	4.62 (1.04-15.34)
	*D*_2/3_(Gy_3_)	1.94 (0.35-2.32)	1.32 (0.62-5.40)	3.55 (1.00-10.87)	3.03 (0.85-28.00)
	*V*_5_(cm^3^)	13.98 (0-87.7)	3.87 (0-70.76)	42.17 (0-98.94)	28.13 (0-94.16)
	*V*_10_(cm^3^)	0.76 (0-38.35)	0 (0-28.79)	9.34 (0-71.45)	5.12 (0-63.29)
Right Kidney	*D*_mean_(Gy_3_)	1.93 (0.87-9.26)	1.37 (0.90-7.27)	3.51 (2.24-16.07)	2.68 (1.70-13.87)
	*D*_2/3_(Gy_3_)	1.40 (0.67-4.93)	1.01 (0-4.70)	2.66 (1.49-8.25)	2.11 (1.24-7.06)
	*V*_5_(cm^3^)	2.03 (0-65.04)	0 (0-60.87)	10.49 (0.11-87.23)	3.90 (0-79.71)
	*V*_10_(cm^3^)	0 (0-29.18)	0 (0-20.95)	0.61 (0-53.16)	0 (0-44.12)
Liver	*D*_mean_(Gy_3_)	3.22 (0.45-8.72)	1.89 (0.69-8.23)	5.35 (1.18-12.49)	4.28 (1.00-10.72)
	*D*_1/2_(Gy_3_)	2.13 (0.35-8.21)	1.25 (0.40-6.67)	3.76 (0.87-11.43)	3.03 (0.57-9.53)
	*V*_10_(cm^3^)	3.08 (0-35.31)	0.21 (0-28.1)	10.93 (0.03-55.26)	3.42 (0-47.67)
	*V*_30_(cm^3^)	0 (0-1.60)	0 (0-0.88)	0.06 (0-3.86)	0 (0-2.66)

### Toxicity

Eighteen patients experienced grade 2 or more adverse events. Among these patients, one patient had grade 3 vomiting as an acute gastrointestinal toxicity and one patient had grade 3 gastrointestinal bleeding as a late toxicity. They all recovered after the treatment. The radiation doses to the stomach, duodenum, and bowel of these two patients were extracted and compared with the median summed does of those OARs (**Table 4**). As a result, most of the doses to the OARs of these two patients were higher than the median accumulated doses.

**Table 4:** Comparisons of doses to OARs of patients with grade 3 toxicity and median summed doses.

		Median summed dose*	Summed dose (case 1)*	Summed dose (case 2)*
Stomach	*D*_max_(Gy_3_)	36.75	33.93	43.46
	*D*_1cc_(Gy_3_)	27.86	27.86	35.07
	*D*_10cc_(Gy_3_)	19.78	21.95	27.36
	*D*_mean_(Gy_3_)	6.42	9.19	10.98
	*V*_10_(cm^3^)	66.41	157.73	160.69
	*V*_20_(cm^3^)	7.69	16.55	39.01
	*V*_30_(cm^3^)	0.40	0.33	5.35
Duodenum	*D*_max_(Gy_3_)	30.36	45	39.21
	*D*_1cc_(Gy_3_)	22.13	39.85	32.3
	*D*_5cc_(Gy_3_)	15.99	37.96	23.41
	*D*_10cc_(Gy_3_)	12.82	35.69	20.84
	*D*_mean_(Gy_3_)	6.18	15.94	9.91
	*V*_10_(cm^3^)	18.66	151.23	60.39
	*V*_20_(cm^3^)	1.72	56.03	12.33
	*V*_30_(cm^3^)	0.05	4.2	0.54
Intestine	*D*_max_(Gy_3_)	35.76	38.1	43.1
	*D*_1cc_(Gy_3_)	28.06	31.14	33.78
	*D*_5cc_(Gy_3_)	22.54	28.79	31.5
	*D*_mean_(Gy_3_)	4.03	9.92	8.92
	*V*_20_(cm^3^)	8.56	33.34	43.89
	*V*_30_(cm^3^)	0.30	0.59	3.03

*All of the summed doses were calculated based on the correlation between dose attenuation and time interval.

Due to low incidences of grade 3 gastrointestinal toxicity, grade 2 adverse effects were included for the identification of potential predictors. After multivariate analysis, the *V*_10_ of the stomach [hazard ratio (HR): 1.02 (95% CI: 1.00–1.04), P = 0.035] and *D*_mean_ of the intestine [HR: 1.78 (95% CI: 1.00–3.18), P = 0.049] correlated with grade 2 or more gastrointestinal toxicity (**Table 5**).

**Table 5:** Factors predictive of grade 2 or more gastrointestinal toxicity.

OAR	Dose	Univariate analysis		Multivariate analysis	
		HR (95% CI)	P value	HR (95% CI)	P value
Stomach	*D*_max_(Gy_3_)	1.04 (0.99-1.09)	0.081	NA	NA
	*D*_1cc_(Gy_3_)	1.06 (0.99-1.21)	0.080	NA	NA
	*D*_10cc_(Gy_3_)	1.16 (1.03-1.31)	0.016	NA	NA
	*D*_mean_(Gy_3_)	1.66 (1.16-2.38)	0.006	NA	NA
	*V*_10_(cm^3^)	1.02 (1.01-1.04)	0.006	1.02 (1.00-1.04)	0.035
	*V*_20_(cm^3^)	1.10 (1.02-1.20)	0.018	NA	NA
	*V*_30_(cm^3^)	1.24 (0.87-1.76)	0.228	NA	NA
Duodenum	*D*_max_(Gy_3_)	1.01 (0.96-1.07)	0.691	NA	NA
	*D*_1cc_(Gy_3_)	1.04 (0.96-1.13)	0.311	NA	NA
	*D*_5cc_(Gy_3_)	1.08 (0.98-1.19)	0.142	NA	NA
	*D*_10cc_(Gy_3_)	1.10 (0.98-1.23)	0.114	NA	NA
	*D*_mean_(Gy_3_)	1.17 (0.96-1.43)	0.130	NA	NA
	*V*_10_(cm^3^)	1.04 (0.99-1.08)	0.107	NA	NA
	*V*_20_(cm^3^)	1.09 (0.94-1.26)	0.258	NA	NA
	*V*_30_(cm^3^)	1.49 (0.67-3.30)	0.324	NA	NA
Intestine	*D*_max_(Gy_3_)	0.99 (0.92-1.06)	0.676	NA	NA
	*D*_1cc_(Gy_3_)	1.04 (0.94-1.15)	0.415	NA	NA
	*D*_5cc_(Gy_3_)	1.07 (0.95-1.22)	0.263	NA	NA
	*D*_mean_(Gy_3_)	2.08 (1.18-3.67)	0.012	1.78 (1.00-3.18)	0.049
	*V*_20_(cm^3^)	1.06 (1.00-1.11)	0.050	NA	NA
	*V*_30_(cm^3^)	1.20 (0.84-1.73)	0.319	NA	NA

### Prediction of grade 2 or more gastrointestinal toxicity

**Figure 3:**
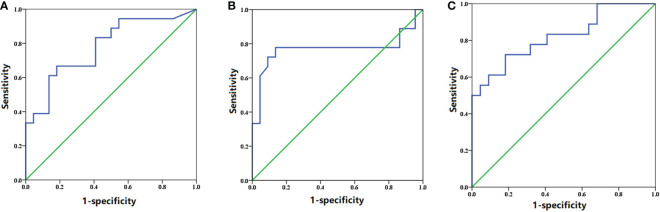
ROC curve of the **(A)** *V*_10_ of the stomach, **(B)** *D*_mean_ of the intestine, and **(C)** combination of the two factors.

After multivariate analysis, the equation was as follows: $\text{P}=\frac{1}{1+e^{-\left(-4.155+0.579X1+0.021X2\right)}}$ X1 = *D*_mean_ of the intestine, X2 = *V*_10_ of the stomach. The value of the goodness of fit of the model derived from the *V*_10_ of the stomach and *D*_mean_ of the intestine was 0.514, which was better than that of the model from each one (*V*_10_ of the stomach: 0.376, *D*_mean_ of the intestine: 0.067). In addition, the threshold and area under the curve (AUC) of the *V*_10_ of the stomach were 77.575 cc and 0.779, while the threshold and AUC of the *D*_mean_ of the intestine were 4.22 Gy_3_ and 0.769, respectively (**Figures 3A, B** **)**. Based on the probability of toxicity of each patient from logistic analysis with the two factors, further analysis with ROC curves showed that the threshold of probability of grade 2 or more gastrointestinal toxicity if patients receive the doses above the threshold of the *D*_mean_ of the intestine and *V*_10_ of the stomach was 0.4345 and the AUC was 0.821 (**Figure 3C**).

## Discussion

Due to the high dose per fraction of SBRT, even a small geometric inaccuracy or uncertainty after image registration could potentially reduce the therapeutic ratio and lead to radiation-induced toxicity. However, direct dose summations with the RIR may contribute to the inaccurate delivery of SBRT due to different patient postures, tumor growth, or gastrointestinal motility. The employment of the DIR technique may provide the potential to obtain more realistic plan sums. So far, it has been previously demonstrated that the DIR had been investigated in dose accumulations in head and neck tumor (27, 28), thoracic tumor (29, 30), and pelvic tumor (31–33). However, no studies have focused on dose summations of re-irradiation with SBRT in pancreatic cancer. Therefore, in this pilot study, the propagation of OAR contouring and transferring of dose distributions were performed for comparisons between standard dose constraints in TG-101 and accumulated doses and evaluations of correlations of radiation-induced gastrointestinal toxicities and dose distributions of OARs from the two treatment plans.

**Figure 4:**
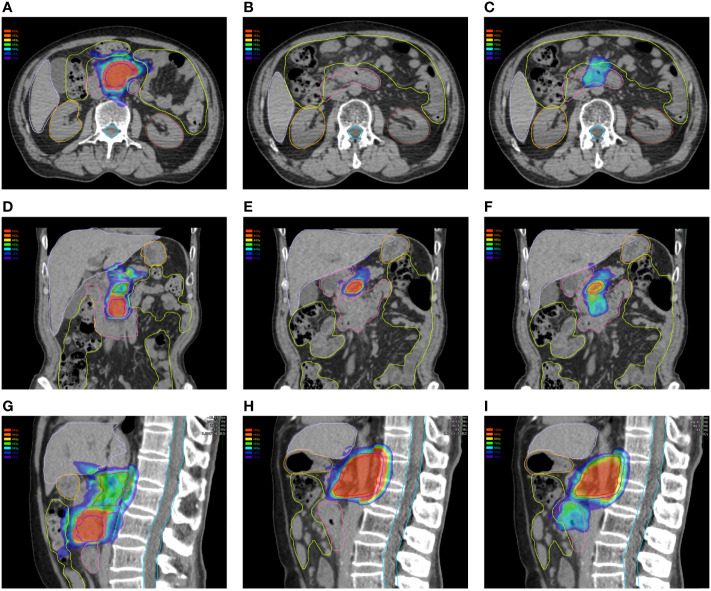
Dose distributions of the first plan in the **(A)** horizontal plane, **(D)** coronal plane, and **(G)** sagittal plane. Dose distributions of the first plan in the **(B)** horizontal plane, **(E)** coronal plane, and **(H)** sagittal plane. Dose distributions of the first plan projected to the second CT scans in the **(C)** horizontal plane, **(F)** coronal plane, and **(I)** sagittal plane.

In this study, the dose–volume parameters of each OAR at the first and second SBRT were all below the corresponding standard dose constraints. However, some of the accumulated dose parameters to the stomach, duodenum, and intestine surpassed the dose constraints without consideration of heal assumption, while doses to the spinal cord, kidneys, and liver far from the target volume were all lower than the dose constraints. Even if dose downscaling due to the time interval between the two courses was taken into account, there were still some but fewer dose parameters above the dose constraints, which might be attributable to gastrointestinal toxicity. Additionally, further analyses on the dose distributions and OAR contouring regarding patients with accumulated doses above the dose constraints were performed. We found significant displacement of the stomach and duodenum in three patients at the second SBRT compared with the first one due to tumor shrinkage after the first treatment. Therefore, some of the dose distributions in the target volume at the first SBRT were projected to the OAR at the second SBRT (**Figure 4**), which resulted in the accumulated doses of OAR above the direct summation of the first and second doses. This error may be ascribed to the failure to compensate for the displacement of OARs due to the significant changes of the tumor volume with the DIR. This was one of the limitations of the DIR known as tissue appearance or disappearance (TAD) (29). Additionally, TAD was also common in the image registration when the second images were taken after surgery, which led to significant anatomical changes between the two images. Actually, TAD has not been taken into consideration in the deformation models of the DIR. Continuity, smoothness, or diffeomorphism may be considered during image registrations in the case of the underlying assumption used to model the deformations. However, these factors were different from TAD. The displacement field abutting to the TAD was distorted resulting in inaccurate accumulated dose distributions. Therefore, several frameworks had been proposed. Nithiananthan et al. (34) had proven that the Demons deformable registration process to include segmentation and an extra dimension in the deformation field could accommodate missing tissues between image acquisitions. Another study also provided a non-rigid registration framework for accommodation of resection and retraction (35). Nevertheless, it still remained a problem during the performance of the DIR, and adoption of dose accumulations in the case of TAD should be taken with caution.

Moreover, dose attenuations between the two courses of radiotherapy were also a challenge for dose prescriptions at re-irradiation and image registrations. The radiobiological rationale for heal assumption between different time intervals has been rarely investigated. In terms of re-irradiation, the summation of doses from different dose-fractionation schedules remained controversial, although normal tissue response might be predicted with the linear-quadratic (LQ) model (36). However, the role of the LQ model in predicting the normal tissue complication probability (NTCP) was limited because this model was derived from survival assays of cancer cell lines *in vitro*. Therefore, dose distributions of normal tissues *in vivo* could not be imitated well with the LQ model. Moreover, the optimal α/β ratio for each normal tissue was unknown. So far, only Abusaris et al. (19) reported the potential correlation between dose downscaling and time periods but without biological evidence when performing dose summation and evaluation of toxicity after re-irradiation for lung tumors. It was proposed in their study that 25% and 50% heal assumption could be estimated 6–12 months and 12 months after radiotherapy, respectively. Similarly, Meijneke et al. (37) recalculated all doses from different plans based on the EQD_2_. Hence, the results in the pilot study should be extrapolated in clinical practice with great caution, which needs to be further validated. Additionally, many relevant factors, in addition to dose distributions and time intervals, should be taken into account in the case of assessment of doses to normal tissues, including the expected survival, curative or palliative intent, OARs overlap with or adjacent to target volumes, and anticipated NTCP based on detailed dosimetry from two or more schedules.

Additionally, it was elucidated in the study that the *V*_10_ of the stomach and *D*_mean_ of the intestine were predictors of grade 2 or more gastrointestinal toxicity. The derived thresholds indicated a lower risk of adverse effects with the *V*_10_ of the stomach below 77.575 cc and *D*_mean_ of the intestine below 4.22 Gy_3_. Furthermore, combined with these two factors, the equation demonstrated the probability of toxicity. Also, the threshold of the probability based on the probability of each patient having toxicity from logistic analysis with the two factors implied that the risk of radiation-induced severe gastrointestinal toxicity could be increased in the event of the probability above 0.4345. So far, previous studies only focused on gastrointestinal dose tolerance at the first SBRT. However, due to the high incidence of local recurrence of pancreatic cancer, SBRT has been employed in the re-irradiation of the local progression with good local control and mild toxicity (8, 16, 17). Therefore, it was required that evaluations of dose distributions of OARs should be given the first priority at the re-irradiation, albeit no investigations had been performed. Compared with previous studies, the thresholds of the stomach and intestine dose–volume were higher. The underlying reason may be attributable to the residual doses to the OARs from the first SBRT. Combined with the two factors, the AUC was larger than that of each one alone. Therefore, the threshold of probability from two factors by logistic analysis may be more accurate in the prediction of toxicity. Great attention should be placed when the probability of severe gastrointestinal toxicity was above the threshold calculated from the equation.

Nevertheless, there were some limitations in the study. The first one was that no radiobiological model could precisely predict the dose downscaling after the first SBRT. Also, owing to the failure to accommodate the TAD in the DIR, the accumulated doses may not be as accurate as the actual ones. Therefore, the clinical practice of the dose thresholds of the stomach and intestine as dose constraints at the re-irradiation, the equation, and the threshold probability of the gastrointestinal toxicity should be taken with great caution. Another one was that the equation and the thresholds have not been internally and externally validated because of the limited number of patients. Additionally, due to careful evaluations of patients in re-SBRT to reduce the risk of severe adverse events, we could not deliver a high radiation dose; therefore, few grade 2 or more toxicities were observed. Third, due to interoperator variability in contouring the intestine, the *D*_mean_ of the intestine may vary between physicians. This might result in the overestimation or underestimation of the risk of gastrointestinal toxicity. Therefore, the interpretation of the equation should be done cautiously. However, compared with previous studies about re-irradiation with SBRT for pancreatic cancer, the number in this study was relatively large.

In conclusion, this pilot study demonstrated that the *V*_10_ of the stomach and *D*_mean_ of the intestine correlated with severe gastrointestinal toxicity after two courses of SBRT. The prediction of gastrointestinal toxicity may be more accurate with these two factors compared to each one alone. Additionally, a higher risk of toxicity may be found in patients with a *V*_10_ of the stomach above 77.575 cc or *D*_mean_ of the intestine above 4.22 Gy_3_ or the probability above 0.4345. Nevertheless, these thresholds and the equation should be further validated.

## Data availability statement

The raw data supporting the conclusions of this article will be made available by the authors, without undue reservation.

## Ethics statement

The studies involving human participants were reviewed and approved by Changhai Hospital. The patients/participants provided their written informed consent to participate in this study.

## Author contributions

HZ was supervised the study. YC, CY and YS designed the treatment plans. XZ, LJ and XG performed patients’ follow-up. XZ analyzed data. YC, XZ and CY drafted the manuscript. HZ revised the manuscript. All authors contributed to the article and approved the submitted version.
